# Perception of Medical Students and Faculty Regarding the Use of Artificial Intelligence (AI) in Medical Education: A Cross-Sectional Study

**DOI:** 10.7759/cureus.77514

**Published:** 2025-01-15

**Authors:** Sudha Rani, Anita Kumari, Shreyasi C Ekka, Ratnajeet Chakraborty

**Affiliations:** 1 Anatomy, Sheikh Bhikhari Medical College, Hazaribagh, IND; 2 Physiology, Sheikh Bhikhari Medical College, Hazaribagh, IND; 3 Orthopedics, Rajendra Institute of Medical Sciences, Ranchi, IND

**Keywords:** artificial intelligence (ai), healthcare technology, medical education, medical student perspectives, medical training

## Abstract

Introduction

Artificial intelligence (AI) is revolutionizing healthcare, offering opportunities to improve diagnosis, clinical care, and medical education. Despite its growing importance, familiarity with AI in medical education remains limited, necessitating a deeper understanding of perceptions among medical students and faculty. The aim of the study is to explore the perceptions of medical students and faculty regarding the use of AI in medical education and its implications for curriculum improvement.

Materials and methods

A cross-sectional study was conducted over six months (January-June 2024) at Sheikh Bhikhari Medical College, Hazaribagh, India. An online questionnaire was distributed to 299 participants, including 242 (80.93%) students, 20 (6.68%) residents, and 37 (12.04%) faculty members, using convenience sampling. Data was analyzed using IBM SPSS Statistics for Windows, Version 26 (Released 2019; IBM Corp., Armonk, NY, USA).

Results

The results revealed that 260 (86.95%) understood AI concepts, but only 36 (12.04%) were very familiar with its application in education. Additionally, 260 (87%) supported AI integration into medical curricula, and 273 (91.3%) believed it could improve educational efficiency. However, 179 (59.9%) had no prior experience with AI tools. Participants highlighted AI's potential in diagnostics (154, or 51.5%), clinical reasoning (51, or 17.1%), radiology (50, or 16.7%), pathology (31, or 10.4%), and 265 (88.62%) expressed a desire for structured AI training.

Discussion

While enthusiasm for AI integration is evident, gaps in exposure and structured education persist. Similar findings in global studies underline the urgent need for standardized curricula and faculty training.

Conclusion

This study highlights the importance of incorporating AI in medical education to prepare healthcare professionals for future challenges. Addressing gaps in knowledge and providing practical exposure are crucial for leveraging AI's full potential in medicine. Further multi-center studies are recommended to validate these findings.

## Introduction

The ability of machines to imitate human behavior is known as artificial intelligence (AI) [1]. It is a collection of mathematical models, represented as algorithms, that can quickly and efficiently learn and analyze vast amounts of data in a variety of formats. Among its many other uses in the medical field, it can help improve the precision and speed of diagnosis, expedite and simplify clinical care, and support public health initiatives [2,3]. Even though these ideas are relatively new, the sheer volume of healthcare data being generated and its rapid digitization mean that AI is quickly becoming a new reality in medical practice [4].

Global healthcare systems are predicted to undergo a change due to AI. In addition to the financial advantages, AI is anticipated to improve healthcare efficiency for patients and healthcare providers. AI has the potential to alleviate the shortage of healthcare workers by analyzing and learning from computer data, mimicking human thought processes. AI enhances the ability to store and search medical data, providing decision-support mechanisms that are transforming the future of healthcare [5].

AI can be utilized in clinical diagnosis, especially in regions facing a shortage of medical doctors. AI can also be used to decrease human error in image processing in radiology and histopathology. Additionally, it can be used to interpret electrocardiograms (ECGs) in cases of atrial fibrillation, ventricular tachyarrhythmias, and myocardial infarction [6]. AI can also be applied to detect sleep disorders and epilepsy, analyze electromyography (EMG) data, and conduct Doppler ultrasound assessments for patients in Intensive Care Units (ICUs) [7-9].

AI integration in medical education is a quickly developing topic that could have an impact on both teachers and students. This study was presented at an International Physiology Conference with the theme Clinical and Interventional Physiology: Current & Future Scope in the Indian Scenario, organized by the Department of Physiology, AIIMS Patna. The purpose of this cross-sectional study is to investigate how teachers and medical students see the application of AI in medical education. The findings ought to serve as the cornerstone for enhancing medical student education and the curriculum.

## Materials and methods

This study was done by using an online questionnaire-based survey, among Jharkhand medical students and faculty members. This is a cross-sectional study, conducted in the Department of Physiology, Sheikh Bhikhari Medical College, Hazaribagh, India.

The convenience sampling method was used to choose the sample population. The period of study was six months, from January 2024 to June 2024. Google Docs was used to compile the questionnaire, and the WhatsApp app and messages were used to distribute the forms to medical students and faculty members via their social networks. The questionnaire was developed based on general principles or guidelines derived from the literature review. Three independent investigators developed the questionnaire, and the discrepancy was resolved by discussion. To evaluate the technical functionality and usefulness of the online questionnaire, a pilot study comprising 30 medical students was carried out. The survey questionnaire was revised by an academic team that consisted of one professor and two associate professors from the Department of Community Medicine and Pathology of Sheikh Bhikhari Medical College. Two months were allotted for this process; no personal information was gathered or stored, and two to four reminders were sent. The survey questionnaire consisted of two sections. The first section entailed their demographic information (age, gender, level of qualification, year of experience in the job). The second portion of the survey questionnaire includes knowledge, perception of AI, and its application in medical education. The data was cleared of duplicate entries. Every participant was made aware of the purpose and design of the study, their freedom to withdraw at any time, and that the information would be gathered anonymously. The voluntary involvement of Jharkhand Medical College staff and students served as the study's inclusion criteria. People who refused to take part in the study were excluded.

This study was a cross-sectional study, and the participants' anonymity and autonomy were greatly valued. Participants in this study could not be traced because no name or email was included. The study ensured that every participant's privacy was sufficiently safeguarded.

Ethical approval had been taken (Letter No. IEC/05/2024, dated 10/01/2024). The collected data was entered into Microsoft Excel (Microsoft® Corp., Redmond, WA, USA), and statistical analysis was done by using IBM SPSS Statistics for Windows, Version 26 (Released 2019; IBM Corp., Armonk, NY, USA). Duplicate entries and incomplete responses were removed from the data.

## Results

Out of 299 participants, 242 (80.93%) were medical students, 20 (6.68%) were residents, and 36 (12.04%) were faculty members. The male respondents were a little higher (164, or 54.84%) compared to females (135, or 45.15%), and most of them were between the age group of 17 to 30 years (247, or 82.6%). Participant characteristics have been mentioned in Table 1.

**Table 1:** Baseline characteristics of the respondents N represents the number of responses, (total N = 299)

Category	Values, N (%)
Age (years)	
17-30	247 (82.6%)
31-40	16 (5.4%)
41-50	21 (7.0%)
51-60	10 (3.3%)
Above 60	5 (1.7%)
Gender	
Male	164 (54.8%)
Female	135 (45.2%)
Qualification	
Undergraduate	229 (76.6%)
Graduate	12 (4.0%)
Postgraduate	58 (19.4%)
Academic affiliation	
Medical students	242 (80.9%)
Residents	20 (6.7%)
Faculty	37 (12.4%)
Years of experience	
1-2 years	244 (81.6%)
2-3 years	7 (2.3%)
3-4 years	6 (2.0%)
4-5 years	6 (2.0%)
5-6 years	36 (12.0%)

**Figure 1:**
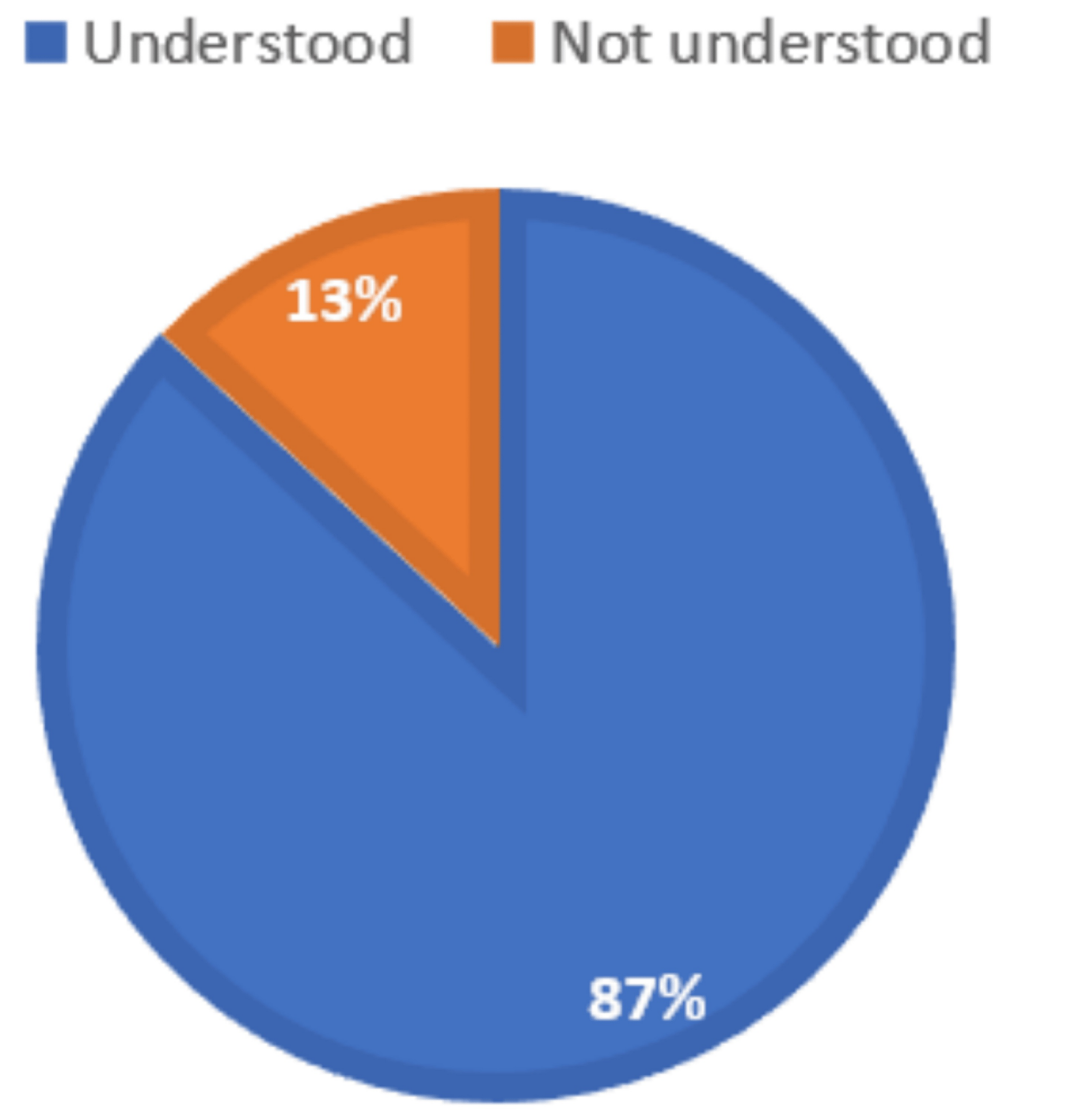
Distribution of respondents according to whether they understood the concept of AI AI, artificial intelligence

**Figure 2:**
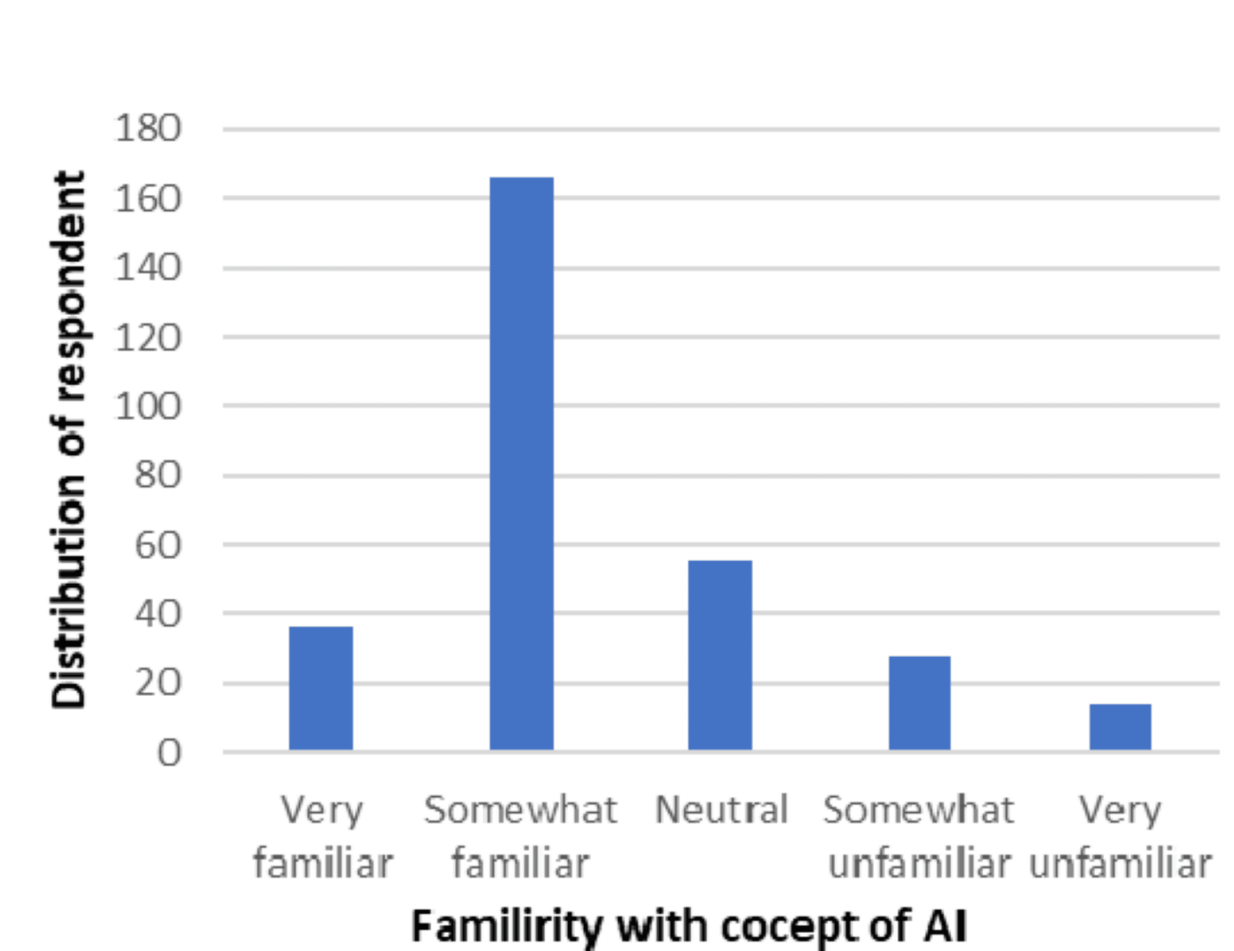
Distribution of respondents according to whether they are familiar with the concept of AI in the context of medical education AI, artificial intelligence

Regarding understanding the concept of AI, the majority of participants (260, or 86.95%) responded yes (Figure 1), but we saw discrepancies in its use in medical education. Only 36 (12.04%) were very familiar, 166 (55.51%) were somewhat familiar, 55 (18.39%) were neutral, 28 (9.36%) were somewhat unfamiliar, and 14 (4.68%) were very unfamiliar (Figure 2).

Table 2 shows that most students and faculty were somewhat comfortable (109, or 36.5%) and very comfortable (67, or 22.4%) with the integration of AI in medical education. Most of the respondents (166, or 55.5%) were somewhat familiar, and 36 (12.0%) were very familiar with the concept of AI in the context of medical education.

**Table 2:** Concept and perception towards use of AI in medical education N represents the number of responses, (total N = 299) AI, artificial intelligence

Category	Values, N (%)
Concept of AI	
Yes	260 (87.0%)
No	39 (13.0%)
Concept of AI in the context of medical education	
Very familiar	36 (12.0%)
Somewhat familiar	166 (55.5%)
Neutral	55 (18.4%)
Somewhat unfamiliar	28 (9.4%)
Very unfamiliar	14 (4.7%)
Comfort with AI in medical education	
Very comfortable	67 (22.4%)
Somewhat comfortable	109 (36.5%)
Neutral	91 (30.4%)
Somewhat uncomfortable	25 (8.4%)
Not comfortable at all	7 (2.3%)
Encountered AI-based tools or resources during medical studies	
Yes	120 (40.1%)
No	179 (59.9%)

**Figure 3:**
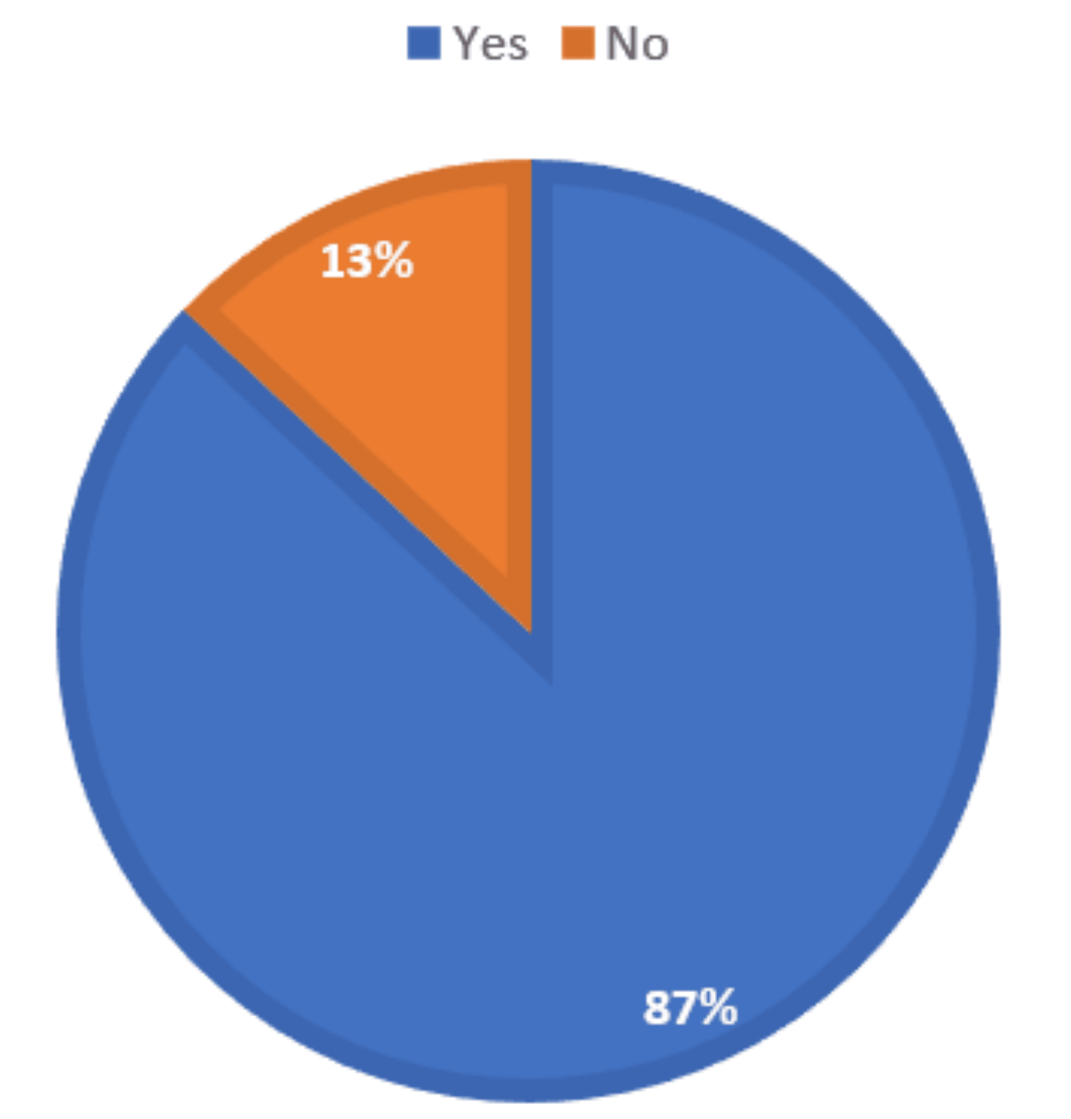
Distribution of respondents according to whether they believe that AI should be part of the medical curriculum AI, artificial intelligence

**Figure 4:**
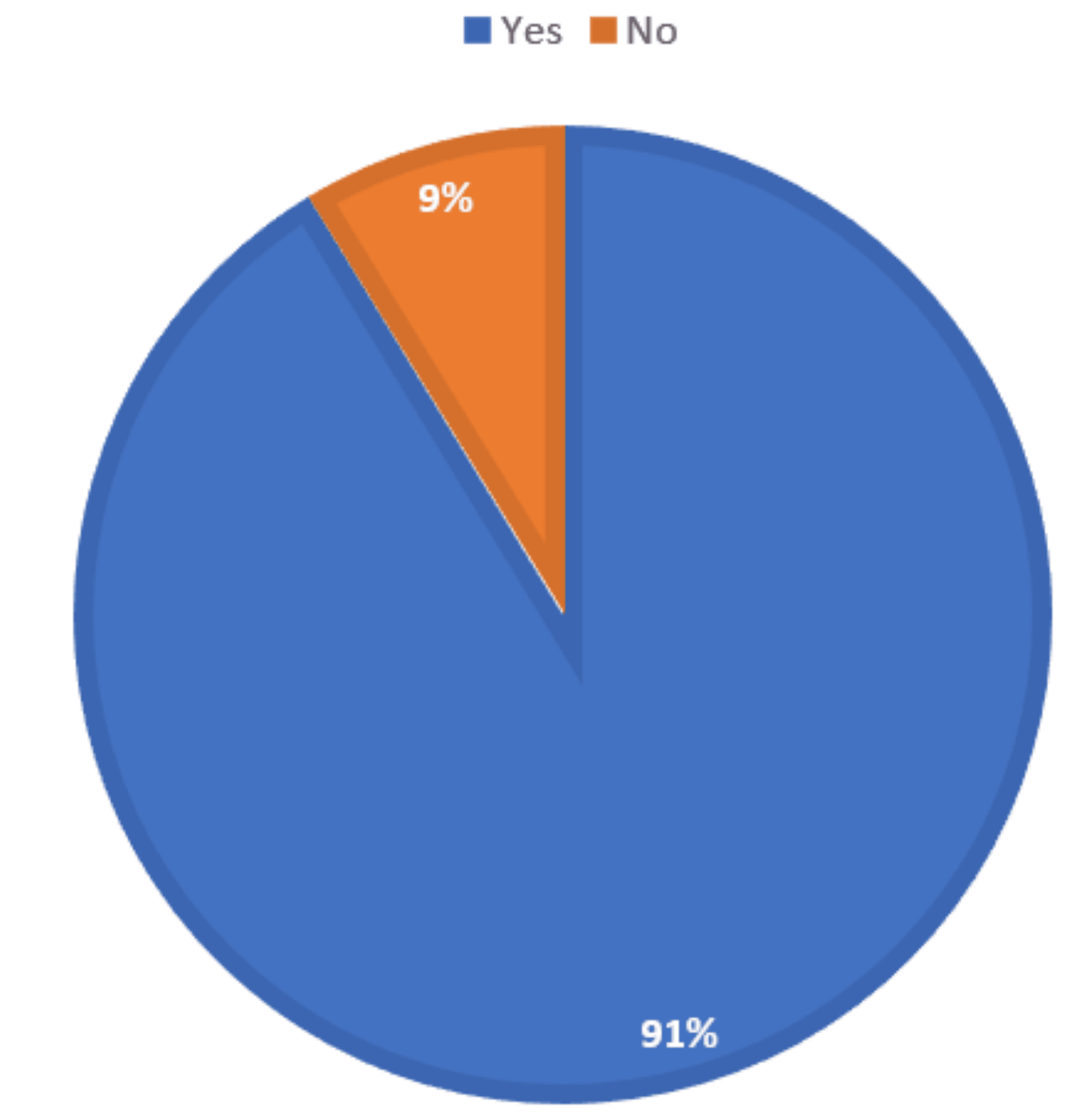
Distribution of respondents according to whether they believe that AI can improve the efficiency of medical education programs AI, artificial intelligence

A total of 179 (59.9%) respondents did not encounter AI-based tools or resources during medical education, but the majority of 260 (87.0%) respondents believe that AI should be part of medical education (Figure 3). In addition, 125 (41.8%) of respondents believe it is effective, 96 (32.1%) somewhat effective, and 47 (15.7%) very effective regarding the use of AI in enhancing the learning experience in medical education. The majority, 273 (91.3%) respondents, also believe that AI can improve the efficiency of medical education (Figure 4).

**Figure 5:**
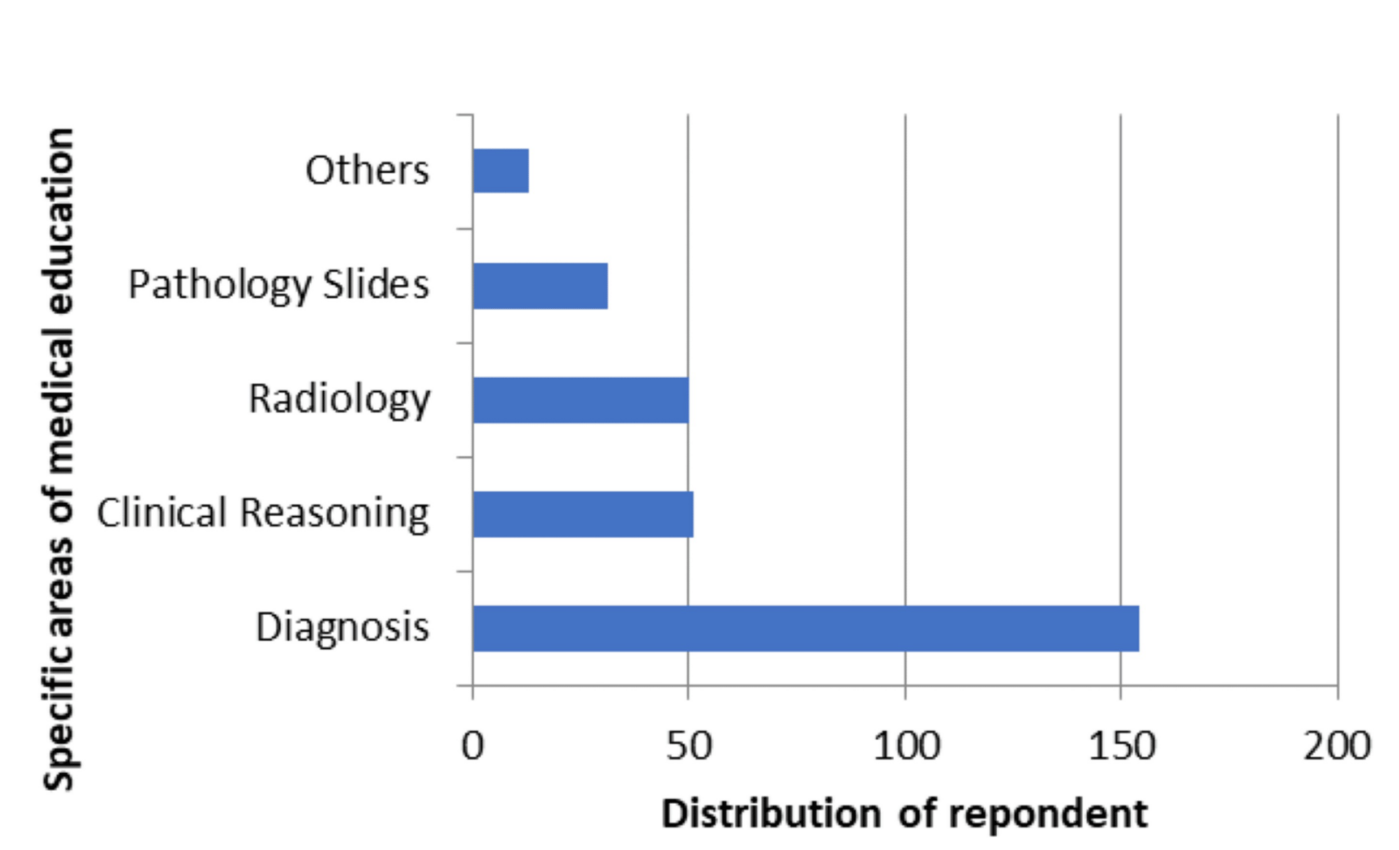
Specific areas of medical education where AI can benefit the most AI, artificial intelligence

Table 3 further illustrates how participants' willingness to employ AI reflects its impact on medical education. According to the majority of participants, integrating AI systems could improve particular areas of medical education, like diagnosis (154, or 51.5%), clinical reasoning (51, or 17.1%), radiology (50, or 16.7%), pathology slides (31, or 10.4%), and other areas (13, or 4.3%) (Figure 5).

**Table 3:** Impact of AI on medical education N represents the number of responses, (total N = 299) AI, artificial intelligence

Category	Values, N (%)
Effectiveness of AI in enhancing the learning experience in medical education	
Very effective	47 (15.71%)
Effective	125 (41.8%)
Somewhat effective	96 (32.1%)
Not effective at all	7 (2.34%)
No opinion	24 (8.02%)
AI should be part of the medical curriculum	
Yes	260 (86.95%)
No	39 (13.04%)
Can AI improve medical education programs	
Yes	273 (91.3%)
No	26 (8.69%)
Specific areas of medical education where AI can benefit the most	
Diagnostics	154 (51.5%)
Clinical reasoning	51 (17.05%)
Radiology	50 (16.72%)
Pathological slides	31 (10.36%)
Other areas	13 (4.3%)
AI can enhance performance in diagnostics and management if included in the medical curriculum	
Yes	249 (83.27%)
No	50 (16.72%)
AI-generated feedback in day-to-day assignments can enhance the learning experience	
Yes	271 (90.63%)
No	28 (9.36%)
Like to be trained in AI for medical education	
Yes	265 (88.62%)
No	34 (11.37%)
Patient data and ethical issues are a concern during AI integration in medical system	
Yes	265 (88.62%)
No	34 (11.37%)

A total of 249 (83.3%) of participants believe that AI can enhance the diagnosis and management of cases if it is included in the medical curriculum. Additionally, 265 (88.62%) of the participants would like training for the implementation of AI in medical education. There is no statistically significant association between the concept of AI and gender, age, or year of experience.

## Discussion

The advancement of medical education will be impacted by the use of AI in the medical industry. According to this survey, the majority of medical faculty members and students have some knowledge about AI in relation to medical education. Our study also shows that 50.6% of males and 61.8% of females have the concept of AI in the context of medical education. However, only 54% of females in the European Union had a positive opinion of AI, compared to 67% of males [10].

Contrary to our study, Pinto Dos Santos et al. [11] demonstrated that, when it came to the advantages of AI in medical practices, men were more enthusiastic, confident, and had a more favorable attitude.

AI has recently attracted considerable attention in the healthcare industry and is becoming a vital element in its future. It can be used in many different fields, such as medical devices, image and scan analysis, pharmaceuticals, and health informatics [12].

The vast majority of the teachers and students thought AI would be significant in certain areas of medical education. More than 75% of medical students in two studies - one conducted in the United States of America (USA) and the other in the United Kingdom (UK) - thought AI would have a modest to significant impact on medicine during their careers [13,14].

In addition, we found that the faculty and students would also like to be trained on the ethical issues that may arise due to AI applications. This assumption is in line with the query posed by Grunhut et al.: "How can a doctor who is not trained in the field of AI expect to handle ethical situations, such as when a computer algorithm predicts a high likelihood of death for a patient?" [15]. Any educational endeavor must include the knowledge and abilities to prevent and resolve ethical issues, since AI in medicine will unavoidably bring up new ethical issues in addition to the old ones.

Our study showed that medical students and faculty members had basic knowledge about AI but lacked detailed knowledge about its application in medical education. They also have favorable opinions and attitudes toward using AI in healthcare and medical education. Thus, this study provides insights into the readiness and potential barriers to incorporating AI into the medical curriculum. This is vital for educational institutions aiming to stay ahead in training competent healthcare professionals. The study highlights the importance of equipping medical students and faculty with AI-related knowledge and skills, ensuring they are prepared to leverage AI tools effectively in their practice. This aligns with the ongoing shift toward personalized and data-driven healthcare. The study can also inform policymakers and educational institutions about the current state and future needs in medical education regarding AI.

This research has various limitations. Firstly, the sample size was limited. Secondly, the findings reflect the personal views of the participants. Thirdly, the study was dependent on the characteristics of students and faculty members who volunteered, which may have introduced selection bias. Fourthly, the study used self-reported questionnaires, which may have resulted in exaggerated answers about respondents' knowledge of AI and perceived preparedness to integrate it into medical education. Additionally, most of the respondents were young students. Finally, measuring the validity and reliability of the questionnaire with a large sample of students and faculty members from different medical colleges across India is suggested for future studies, as the current study had a limited sample size. Furthermore, conducting multi-center studies with standardized protocols across different medical colleges can improve the generalizability of results.

## Conclusions

Today's medical students will work in a very different environment than the one they are used to as AI systems become more common in the field. The recent pandemic has accelerated this transformation, highlighting the urgent need for changes in medical education and healthcare service delivery. However, students often lack a structured or standardized education on AI, leaving them feeling uninformed and inadequate. Our study investigates how students evaluate AI's possible effects on medicine and the field, as well as what subjects they believe ought to be covered in medical school. Medical students and faculty are eager to learn how to utilize AI technologies while addressing ethical concerns related to AI. This enthusiasm presents a valuable opportunity to train faculty and medical students in AI. We think that the moment is right to reach an agreement on the fundamental knowledge and abilities needed for AI, as well as to use cutting-edge teaching methods to foster AI competency and ethics.

## Appendices

Questionnaire

1) How old are you?

a) 17 to 30 yr

b) 31 to 40 yr

c) 41 to 50 yr

d) 51 to 60 yr

e)> 60 yr

2) What is your gender?

a) Female

b) Male

3) What is your qualification level?

a) Undergraduate

b) Graduate

c) Postgraduate

d) Others

4) What is your academic affiliation?

a) Medical student

b) Resident

c) Faculty

d) Others

5) How much experience do you have in your job?

a) < 1 yr

b) 1 to 2 yr

c) 3 to 4 yr

d) 4 to 5 yr

e) > 6 yr

6) Do you understand the concept of Artificial intelligence?

a) Yes

b) No

7) How familiar are you with the concept of Artificial Intelligence in the context of medical education?

a) Somewhat familiar

b) Very familiar

c) Neutral

d) Somewhat unfamiliar

e) Very unfamiliar

8) How comfortable are you with the integration of AI in your medical education?

a) Somewhat comfortable

b) Very comfortable

c) Neutral

d) Somewhat uncomfortable

e) Not comfortable at all

9) Have you encountered any AI-based tools or resources during your medical studies?

a) Yes

b) No

10) In your opinion, how effective is AI in enhancing the learning experience in medical education?

a) Somewhat effective

b) Effective

c) Very effective

d) Not effective at all

e) No opinion

11) Do you believe that AI should be part of the medical curriculum?

a) Yes

b) No

12) Do you believe that AI can improve the efficiency of medical education programs?

a) Yes

b) No

13) What specific areas of medical education do you think AI could benefit the most?

a) Diagnostics

b) Clinical reasoning

c) Pathological slide

d) Radiology

e) Others

14) Do you want to get trained in artificial intelligence for medical education?

a) Yes

b) No

15) How often have you used AI in your daily life?

a) Daily

b) Once a week

c) Once a month

d) Never

16) Do you think AI can enhance your AI can enhance your performance in diagnostics and management if included in the medical curriculum?

a) Yes

b) No

17) Are there any ethical considerations you believe should be addressed regarding the integration of AI in medical education?

a) Yes

b) No

18) Would you be open to participating in AI-assisted learning experiences as part of your medical education curriculum?

a) Yes

b) No

19) Do you think AI-generated feedback in day-to-day assignments can enhance your learning experience?

a) Yes

b) No

20) Do you think the privacy of patient data and ethical issues are a concern during the integration of AI in the medical system?

a) Yes

b) No
